# Clinical Performance and Optical Quality of a Non-Diffractive Extended-Depth-of-Focus Intraocular Lens in Patients Undergoing Cataract Surgery or Refractive Lensectomy

**DOI:** 10.3390/jcm14113717

**Published:** 2025-05-26

**Authors:** Antonio Cano-Ortiz, Álvaro Sánchez-Ventosa, Timoteo González-Cruces, Marta Villalba-González, Francisco Javier Aguilar-Salazar, Juan J. Prados-Carmona, Vanesa Díaz-Mesa, Alberto Villarrubia

**Affiliations:** 1 Department of Ophthalmology, Hospital Arruzafa, 14012 Cordoba, Spain; 2 Department of Health and Biomedical Sciences, Universidad Loyola Andalucía, 41704 Sevilla, Spain; 3 Department of Ophthalmology, Reina Sofia University Hospital, 14004 Cordoba, Spain

**Keywords:** extended depth of focus, intraocular lens, phacoemulsification, optical quality, cataract

## Abstract

**Objectives:** To evaluate the clinical performance and optical quality of a non-diffractive extended-depth-of-focus (EDOF) intraocular lens (IOL), Asqelio™ EDOF (models ETLIO130C/ETPIO130C), in patients undergoing cataract surgery or refractive lensectomy. **Methods:** This prospective observational, case-control study included patients bilaterally implanted with either the Asqelio™ EDOF IOL (Study Group) or the spherical monofocal TECNIS^®^ 1-Piece ZCB00 IOL (Control Group). The postoperative outcomes—at 3 months after surgery—included visual acuities at multiple distances, refraction, contrast sensitivity, the optical scatter index (OSI), wavefront aberrations, and patient-reported outcomes (Catquest-9SF and a quality-of-vision questionnaire). **Results:** Twenty-three patients (46 eyes) in the Asqelio™ EDOF group and 17 patients (34 eyes) in the monofocal control group were enrolled. Postoperatively, 91% of eyes in the EDOF group were within ±0.50 D of the intended spherical equivalent. The binocular uncorrected distance, intermediate, and near visual acuities were 0.00 ± 0.09, 0.13 ± 0.12, and 0.32 ± 0.15 logMAR, respectively. Contrast sensitivity and OSI values were similar between the study and control groups (*p* > 0.05). Higher-order aberrations were significantly lower in the EDOF group (*p* < 0.001), but values in both groups were clinically low. No adverse events were reported. Most patients expressed high satisfaction and reported few visual disturbances. **Conclusions:** The Asqelio™ EDOF IOL provided good refractive predictability, effective uncorrected vision across distance and intermediate ranges, and high patient satisfaction. Contrast sensitivity and optical scatter were comparable to monofocal implants. This lens can be considered a valuable option for patients seeking an extended range of functional vision with minimal side effects.

## 1. Introduction

Cataract remains one of the leading causes of blindness worldwide, with surgical removal of the opacified lens followed by intraocular lens (IOL) implantation as the definitive treatment. Monofocal IOLs have long been used to correct distance ametropia, albeit with limited near and intermediate visual performance. Bifocal IOLs introduced a second focus for near vision, improving near tasks but often leaving gaps in intermediate vision. In 2012, trifocal IOLs were introduced in Europe, offering improved intermediate vision by providing three distinct focal points, thereby enhancing spectacle independence for a broad range of distances. The additional third focus, specially designed to improve intermediate vision compared to bifocal models, has increased its use among cataract and refractive surgeons aiming to provide their patients better intermediate outcomes. More recently, extended-depth-of-focus (EDOF) IOLs have emerged as an alternative presbyopia-correcting solution. The first EDOF IOL was approved by the U.S. Food and Drug Administration (FDA) in 2016 [1], and subsequent research has demonstrated that EDOF IOLs provide continuous ranges of functional vision, with performance comparable or superior to trifocal designs in intermediate tasks but somewhat less effectiveness at close reading distances [2,3,4]. A meta-analysis found trifocal IOLs to have better near vision performance than EDOF IOLs but at the cost of a slightly higher incidence of photic phenomena [5].

Although several EDOF IOLs have been evaluated in clinical and optical bench studies, evidence regarding the real-world performance of non-diffractive, wavefront-shaping, phase-ring based EDOF lenses remains limited. In particular, while some early clinical data on the Asqelio™ EDOF IOL have been published, there is a lack of comparative studies that directly evaluate its performance against standard monofocal lenses. Given that monofocal IOLs are considered the gold standard for uncorrected distance vision, it is clinically relevant to assess how newer EDOF designs perform in comparison, especially in terms of distance visual acuity, optical quality, and patient satisfaction.

Thereby, the purpose of the present study is to evaluate the optical quality and clinical performance of a non-diffractive, wavefront-shaping, phase-ring-based EDOF IOL, comparing it to a monofocal control lens in patients undergoing cataract surgery or refractive lensectomy. Standard surgical procedures were followed, with 3-month postoperative follow-up.

## 2. Materials and Methods

This study, designed as a prospective, observational, case-control, post-marketing investigation, obtained approval from the Ethics Committee of the Reina Sofia Hospital (Córdoba, Spain). All procedures strictly adhered to the principles outlined in the Declaration of Helsinki. Each participant provided written informed consent prior to enrollment, and the potential benefits and risks of participation were carefully explained. The study was registered at www.clinicaltrials.gov under the identifier NCT06707545.

### 2.1. Study Participants

Patients aged 50 years or older who underwent bilateral cataract surgery were recruited and had received either the spherical monofocal TECNIS^®^ 1-Piece model ZCB00 (Johnson & Johnson Vision, Jacksonville, FL, USA) as the control IOL or the Asqelio™ EDOF model ETLIO130C/ETPIO130C (AST VisionCare, Inc., Billerica, MA, USA) as the study IOL. All patients received care following the usual clinical practice, and both the preoperative assessment and the surgical implantation of IOLs were completed prior to formal study inclusion.

### 2.2. IOL Description

The TECNIS^®^ 1-Piece monofocal IOL model ZCB00 is made from a hydrophobic acrylic material with a refractive index of 1.47 and features an aspheric anterior surface with a negative spherical aberration of −0.27 μm. The implant has a total diameter of 13.0 mm with a 6.0 mm optical zone and includes a square posterior edge around the entire circumference. It is available in a wide range of diopter powers and is intended to improve uncorrected distance vision.

The Asqelio™ EDOF model ETLIO130C/ETPIO130C is a one-piece foldable posterior chamber IOL manufactured via a proprietary Phase-Ring™ technology. It incorporates a non-diffractive, bi-aspheric design for presbyopia correction and includes a spherical aberration of −0.27 μm, a 360-degree sharp edge, and a posterior surface structured with the Phase-Ring™ feature to extend the range of clear vision from distance through intermediate and near. The lens has a total diameter of 13.0 mm and an optical zone of 6.0 mm. It is made from a glistening-free, hydrophobic acrylic soft material with a refractive index of 1.5 and an Abbe number of 50. The EDOF design aims to maintain image quality for distant vision while enhancing intermediate and near performance.

### 2.3. Inclusion and Exclusion Criteria

Eligible patients were required to have age-related cataract prior to surgery, an anticipated postoperative best-corrected visual acuity of 20/25 or better, and transparent intraocular media apart from the cataract. Exclusion criteria comprised preoperative corneal astigmatism exceeding 1.0 D, the withdrawal of informed consent, a history of corneal surgery or trauma, irregular cornea (such as keratoconus), uncontrolled or medically controlled glaucoma, notable macular pathology, corneal dystrophy, non-age-related cataract, advanced optic nerve damage, significant diabetic retinal disease, amblyopia, markedly shallow anterior chambers, active or chronic severe intraocular inflammation, pregnancy or breastfeeding, dense or mature cataracts impeding adequate fundus visualization, a history of retinal detachment, or participation in other ongoing clinical studies. Patients anticipating additional ocular surgery during the study period were also excluded.

### 2.4. Surgical Procedure

A clear corneal incision of 2.2 mm was created, followed by standard phacoemulsification with the Centurion^®^ Vision System (Alcon Labs Inc., Fort Worth, TX, USA). After the lens material was removed and the posterior capsule polished, sodium hyaluronate 1.0% (Provisc™, Alcon) was introduced into the capsular bag. The chosen intraocular lens, either TECNIS^®^ ZCB00 or Asqelio™ EDOF, was then inserted according to standard surgical guidelines. Postoperative treatment involved a tapering regimen of topical moxifloxacin 5 mg/mL (Vigamox™, Alcon), prednisolone 10 mg/mL (Pred-Forte™, Allergan, Irvine, CA, USA), and diclofenac 1 mg/mL (Diplofenac-Lepori) over four weeks.

### 2.5. Variables and Assessments

The pupil size was measured using the IOLMaster 700 (Carl Zeiss Meditec, Jena, Germany). Monocular uncorrected distance visual acuity (UDVA) and best-corrected distance visual acuity (CDVA) were recorded at 4 m under photopic conditions. In the EDOF group, uncorrected and distance-corrected intermediate visual acuity (UIVA and DCIVA) at 67 cm and near visual acuity (UNVA and DCNVA) at 40 cm were also evaluated using the Early Treatment Diabetic Retinopathy Study (ETDRS) chart. Subjective refraction was measured monocularly and binocularly. Contrast sensitivity was determined using the best-corrected Pelli-Robson test, which measures the contrast threshold for a single spatial frequency (20/60 letters) decreasing in 0.15-log-unit steps. For adverse events, both direct patient reports and investigator observations were considered. A binocular defocus curve was acquired in the EDOF group by altering the vergence from −4.0 D to +2.0 D in 0.5 D increments while patients wore their best distance correction. Wavefront aberrations were evaluated using the Hartman–Shack system (Pentacam AXL Wave, OCULUS, Wetzlar, Germany), with particular attention to spherical aberration and coma expressed as Zernike polynomials. The ocular dispersion index (OSI) was measured using a double-pass system (OQAS II, Visiometrics S.L., Terrassa, Spain), where the OSI value is calculated from the proportion of light in an annular region between 12 and 20 arc minutes to that in a circular area within one arc minute around the central peak.

Patient-reported outcomes in the EDOF group were collected through the CATQuest-9SF [6,7], a nine-item questionnaire designed to assess vision-related activity limitations and satisfaction, and a quality of vision questionnaire that evaluates the frequency, intensity, and bothersome nature of ten visual symptoms [8]. For the CATQuest-9SF, responses range from “very great difficulty/very dissatisfied” (score of 4) to “no difficulty/very satisfied” (score of 1), with an additional “cannot decide” option considered as missing data. The quality of vision questionnaire explores specific visual disturbances, such as glare, halos, and starbursts, using a four-point rating for each dimension.

### 2.6. Sample Size and Statistical Analysis

The estimated sample size was determined using G*Power v3.1 (Heinrich Heine Universität, Düsseldorf, Germany), assuming an α of 0.05 and a β of 0.8, with the primary variable being the OSI. Reference values were obtained from analogous studies of comparable IOL designs. Based on these calculations, 24 eyes per group were required. The final sample comprised 23 patients (46 eyes) in the Asqelio™ EDOF IOL group and 17 patients (34 eyes) receiving the TECNIS^®^ 1-Piece monofocal IOL.

Data analysis was conducted on all patients meeting the inclusion criteria and none of the exclusion criteria, using SPSS v25.0 (IBM Corp., Chicago, IL, USA).The level of significance was set at *p* < 0.05. Categorical data are expressed as counts and proportions, while continuous data are reported as mean values with corresponding standard deviations. Cumulative histograms depicting the postoperative refractive error and astigmatism were generated to assess the refractive accuracy, while a cumulative histogram of postoperative visual acuities was created to evaluate the effectiveness of refractive correction. The Shapiro–Wilk test was applied to determine the normality of data distribution; dependent on these outcomes, either the Friedman test or the paired t-test was used for comparisons between groups. All figures presenting refractive and visual outcomes were prepared in accordance with the reporting standards proposed by Reinstein et al. [9].

## 3. Results

A total of 40 patients (80 eyes) bilaterally implanted were enrolled, with 23 in the EDOF group and 17 in the monofocal control group. The mean age for the EDOF group was 64.21 ± 8.24 years (range 47–78), and the mean age for the control group was 73.45 ± 5.87 years (range 63–87). The preoperative spherical equivalent, astigmatism, and axial length were comparable between groups (Table 1).

### 3.1. Refraction

**Figure 1:**
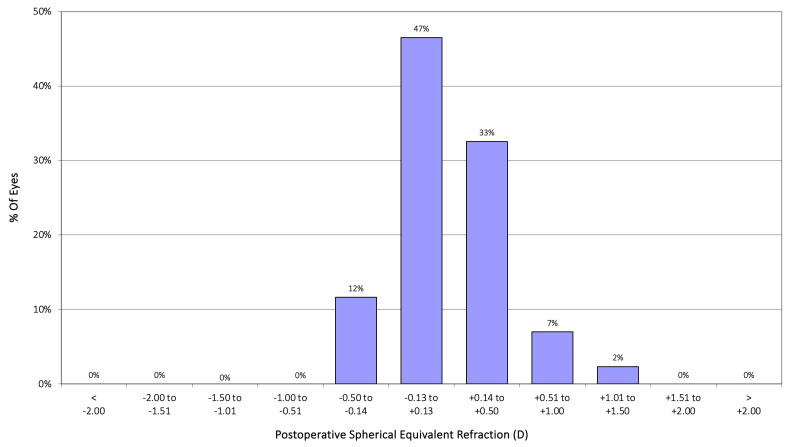
Postoperative spherical equivalent (D) accuracy for the Asqelio™ extended-depth-of-focus intraocular lens.

**Figure 2:**
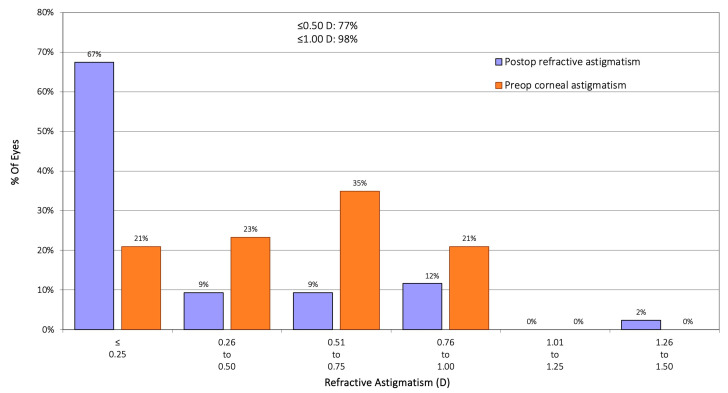
Preoperative corneal astigmatism (D) and postoperative refractive astigmatism (D) for the Asqelio™ extended-depth-of-focus intraocular lens.

**Figure 3:**
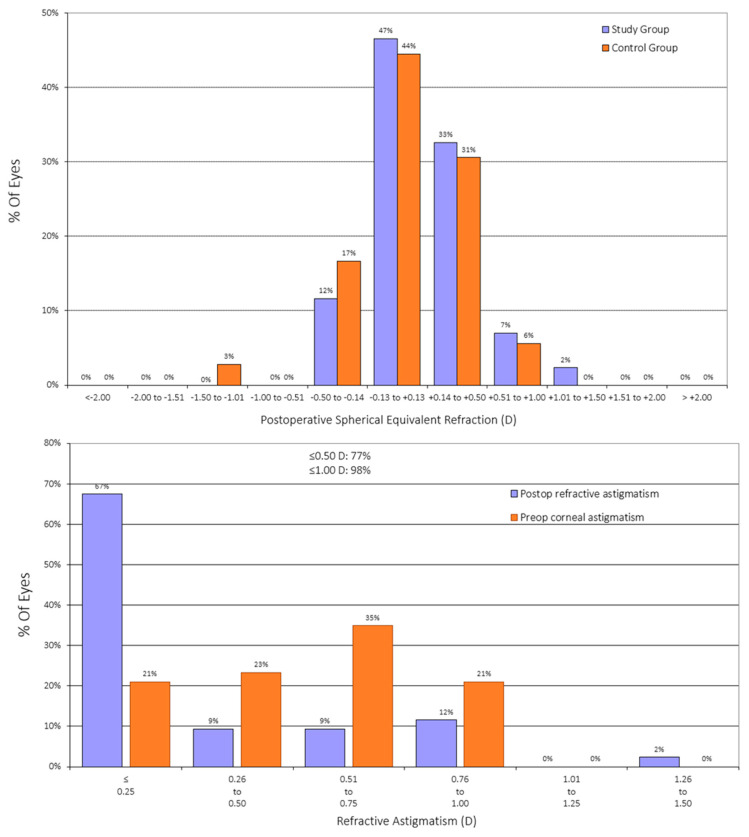
Distribution of postoperative spherical equivalent refraction (**top**) and refractive astigmatism (**bottom**) in the study and control groups.

Figure 1 displays postoperative spherical equivalent accuracy. In the EDOF group, 92% of eyes were within ±0.50 D, and 97% were within ±1.00 D of the intended target. The mean postoperative spherical equivalent was 0.15 ± 0.35 D, not significantly different from the control group (0.09 ± 0.28 D, *p* = 0.214). Postoperative residual astigmatism was ≤0.50 D in 77% of eyes, with a mean refractive cylinder of −0.28 ± 0.41 D, again not significantly different from the control group (*p* = 0.128). For the astigmatism, Figure 2 shows the cumulative histogram representing the postoperative refractive astigmatism against the preoperative corneal astigmatism. A total of 98% of eyes exhibited a postoperative refractive cylinder of ≤1.00 D, and 77% of eyes were within ≤0.50 D. The mean postoperative cylinder was –0.28 ± 0.41 D, with values ranging from 0 to –1.50 D. This residual cylinder value is not significantly different from that obtained in the control group, of −0.36 ± 0.39 D (*p* = 0.128). Figure 3 illustrates that most eyes attained a postoperative spherical equivalent within ±0.50 D and refractive astigmatism ≤0.50 D, demonstrating the procedure’s refractive precision and its efficacy in minimizing residual astigmatism.

### 3.2. Visual Acuity and Defocus Curve

**Figure 4:**
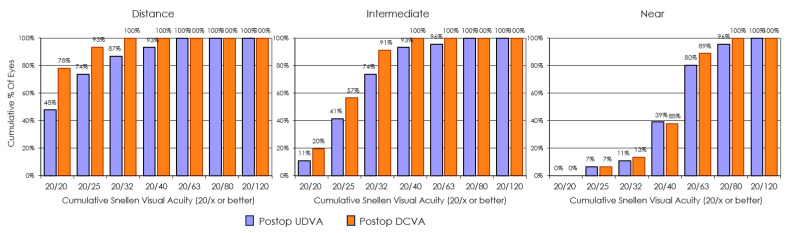
Cumulative postoperative monocular uncorrected distance visual acuity (UDVA) and corrected distance visual acuity (CDVA) at far, intermediate, and near distances in the Asqelio™ extended-depth-of-focus intraocular lens group.

**Figure 5:**
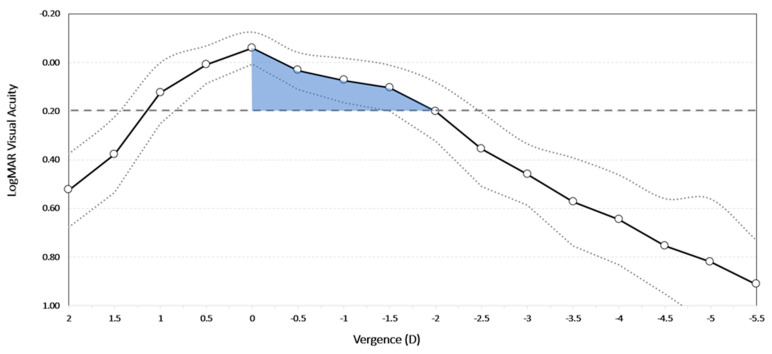
Binocular defocus curve for the Asqelio™ extended-depth-of-focus intraocular lens group. White circles represent mean values, dotted curves represent standard deviation curves, and the dashed line represents the 0.20 LogMAR. The Y-axis shows visual acuity (logMAR), and the X-axis shows vergence (D).

The visual performance of the EDOF IOL implantation is illustrated in Figure 4, where cumulative the postoperative monocular Snellen UDVA is compared with the postoperative monocular Snellen CDVA at far, intermediate, and near distances. Table 2 shows detailed values for the visual acuity at different distances under photopic conditions for the study lens group. The postoperative mean values of the binocular logMAR photopic CDVA, photopic CDIVA, and photopic CDNVA were −0.03 ± 0.08, 0.09 ± 0.08, and 0.29 ± 0.11, respectively. Figure 5 depicts the binocular defocus curves for the EDOF IOL group, showing a broad range of functional vision from distance down to vergences of around 2.25 D. The group × age interaction reached statistical significance (β_3_ = 0.007 logMAR yr^−1^, *p* = 0.005), but we caution that this estimate is based on a cross-sectional comparison at 3 months and should not be extrapolated to future intra-individual change.

On the other hand, no adverse effects impacting postoperative visual acuity were observed. No clinically significant posterior capsule opacification (PCO), cystoid macular edema, IOL decentration, or elevated IOP occurred during follow-up; one control-group eye exhibited trace PCO without a visual impact.

### 3.3. Contrast Sensitivity and Optical Quality

Outcomes for contrast sensitivity and optical quality parameters obtained in both groups are summarized in Table 3. Contrast sensitivity, evaluated using the Peli Robson test, showed similar mean scores in both groups. The difference between the groups was not statistically significant (*p* = 0.386), indicating that the implantation of Asqelio™ EDOF IOL does not negatively affect the ability to distinguish contrasts compared to the monofocal lens. With regards to light scatter values, the OSI is an objective measure of the eye’s optical quality, reflecting the amount of light scattered within the optical system. The observed difference did not reach statistical significance (*p* = 0.160), suggesting that both types of lenses offer similar optical quality in terms of light scattering.

An analysis of higher-order wavefront aberrations (HOARMS) revealed significant differences between the groups. The HOARMS was significantly lower in the EDOF group compared to the control group (*p* < 0.001). Specifically, residual spherical aberration was significantly lower in the EDOF group (*p* < 0.001). The reduction of spherical aberration in the EDOF group suggests that the EDOF IOL improves optical quality by minimizing this type of aberration. The absence of significant differences in contrast sensitivity and OSI indicates that the implantation of the EDOF IOL does not negatively affect key aspects of visual quality compared to monofocal implants.

### 3.4. Patient-Reported Outcomes

A summary of the patient-reported difficulties and satisfaction with their vision (Catquest-9SF) are shown in Table 4, with the summary of patients reporting visual symptoms in Table 5. Seventy percent (70.59%) of EDOF patients reported being “fairly satisfied” or “very satisfied” with their vision after surgery. No patient reported being “very unsatisfied.” Most patients had no difficulty (47.06%) or some difficulty (47.06%) in everyday life. The most commonly reported photic phenomenon was blurred vision or halos, but intensity and bothersomeness remained low. No subject felt significantly impaired by visual disturbances. No adverse events were reported during the 3-month follow-up period.

## 4. Discussion

Such as we have introduced, EDOF IOLs are increasingly being adopted in modern cataract and refractive lens exchange surgeries due to their ability to offer a continuous range of usable vision. Unlike trifocal or bifocal multifocal IOLs that provide distinct focal points for near, intermediate, and distance vision, EDOF IOLs create an elongated focal area that smooths out these transitions. As a result, patients often experience excellent distance and intermediate vision, with reduced visual disturbances compared to multifocal lenses, albeit sometimes at the expense of near vision sharpness [10]. The growing popularity and variety of EDOF IOL platforms have led to significant interest in defining appropriate selection criteria, performance metrics, and clinical endpoints for these lenses. Such efforts prompted the American Academy of Ophthalmology to develop consensus guidelines for EDOF IOLs [11]. In turn, the FDA incorporated these recommendations into the American National Standards Institute (ANSI) Z80.35–2018, thereby creating a standardized benchmark for EDOF lenses [12]. In general, EDOF IOLs should provide a monocular depth of focus at 0.2 logMAR at least 0.5 D greater than a monofocal control and improved intermediate vision without sacrificing distance acuity. Furthermore, at least 50% of eyes should achieve 0.2 logMAR or better VA at 66 cm, and the mean monocular CDVA should remain non-inferior to monofocal IOLs. While parameters such as mesopic contrast sensitivity and visual disturbance questionnaires are required, there are no strict quantitative criteria for these subjective measures. The aim is to balance visual acuity outcomes with the quality of vision and patient satisfaction. Non-diffractive wavefront-shaping EDOF IOL designs, such as AcrySof^®^ IQ Vivity^®^ (Alcon, USA), Lucidis (SAV-IOL, Switzerland), and Asqelio™ EDOF, exemplify the recent advancements in the field. Non-diffractive EDOF IOLs rely on continuous refractive profiles or other wavefront-shaping strategies rather than diffractive gratings. The goal is to minimize haloes, glare, and starbursts typically associated with multifocal lenses, thus improving contrast and patient comfort.

The present study evaluated the clinical outcomes of patients implanted with the Asqelio™ EDOF IOL and compared them to a control group of monofocal lens recipients, with particular focus on the optical quality of the retinal image.

### 4.1. Refractive Outcomes

A key indicator of IOL performance is the predictability of the postoperative refractive outcome. In the current series, the spherical equivalent showed 98% of eyes falling within ±1.00 D and 91% within ±0.50 D of the planned refraction. Nearly half of the eyes (47%) achieved a spherical equivalent outcome within ±0.13 D, and 33% fell between +0.14 and +0.50 D. The mean postoperative spherical equivalent was 0.15 ± 0.35 D, ranging from −0.50 to +1.25 D, closely mirroring the control group’s mean spherical equivalent of 0.09 ± 0.28 D (*p* = 0.214). Thus, the Asqelio™ EDOF IOL demonstrated predictability on par with monofocal lenses. Astigmatism correction outcomes also proved favorable. Ninety-eight percent of eyes achieved ≤1.00 D of astigmatism, and 77% were at ≤0.50 D. The mean postoperative cylinder was 0.28 ± 0.41 D, which was statistically similar to the control group’s 0.36 ± 0.39 D (*p* = 0.128). These results underscore that the EDOF IOL does not inherently introduce higher levels of residual astigmatism compared to a standard monofocal implant. Notably, a subset (26%) of patients exceeded the initial corneal astigmatism inclusion criteria (≥1.00 D). Although these particular eyes were excluded from the monocular analysis, their binocular data were considered.

### 4.2. Visual Acuity Outcomes

In this study, the Asqelio™ EDOF IOL group demonstrated excellent performance across multiple distances. The mean binocular photopic CDVA was −0.03 ± 0.08 logMAR, effectively equal to or better than 20/20 Snellen. UDVA improved markedly from preoperative CDVA levels. Monocular UDVA averaged 0.08 ± 0.13 logMAR (~20/24 Snellen), while binocular UDVA was 0.00 ± 0.09 logMAR, indicating that both eyes together often achieve nearly ideal distance vision. Intermediate vision is a hallmark advantage of EDOF IOLs compared to monofocals. The Asqelio™ implant provided a monocular UIVA of 0.19 ± 0.13 logMAR, which improved binocularly to 0.13 ± 0.12 logMAR. After correcting for distance, CDIVA improved further to 0.09 ± 0.08 logMAR. This intermediate performance is superior to what conventional monofocal lenses typically achieve and aligns with previously reported outcomes using this IOL [13] and other non-diffractive EDoF lenses like AcrySof^®^ IQ Vivity^®^ [14]. UNVA was understandably less robust (0.32 ± 0.15 logMAR binocularly), but the adoption of a mini-monovision strategy, slightly myopic correction in the non-dominant eye, helps bridge the gap. This approach, reported in other studies as well [15,16,17], can significantly enhance near tasks, such as reading fine print, allowing patients to achieve greater spectacle independence. Regarding the initial age difference between groups, the age-dependent slope observed for UDVA likely reflects baseline cohort differences rather than true longitudinal aging, given that follow-up was limited to 3 months. Visual acuity can continue to improve for up to a year after cataract surgery due to neural adaptation and ocular surface stabilization, so this interaction may attenuate or even disappear with a longer follow-up.

### 4.3. Defocus Curve and Depth of Focus

A key EDOF parameter is the defocus curve, which illustrates visual acuity as a function of vergence. In this study, the mean binocular defocus curve revealed a visual acuity peak at 0 D, maintaining good vision through intermediate defocus levels and reaching the 0.2 logMAR threshold at approximately −2.00 D vergence. According to ANSI standards, EDOF IOLs should maintain visual acuity ≤0.2 logMAR over an extended range. The present results confirm compliance with this criterion and match profiles reported in the literature for other EDOF lenses [13,18]. Such performance translates into practical benefits: patients can comfortably perform tasks at computer or dashboard viewing distances without frequent spectacle use.

### 4.4. Contrast Sensitivity and Optical Quality

Contrast sensitivity is essential for functional vision, especially under suboptimal lighting conditions. Using the Pelli–Robson chart, the mean contrast sensitivity scores did not differ significantly between the Asqelio™ EDOF group (1.67 ± 0.10 log units) and the monofocal control group (1.66 ± 0.23 log units; *p* = 0.386). These values are well within normal ranges for age-matched healthy eyes [19]. The preservation of contrast sensitivity is a notable advantage of non-diffractive EDOF designs over many multifocal IOLs that often induce contrast reductions due to splitting light into multiple focal points [20,21]. OSI measurements, an objective indicator of intraocular light scatter, were similarly indistinguishable between EDOF and monofocal groups (*p* = 0.160). This suggests that incorporating EDOF technology does not compromise optical clarity. Moreover, wavefront analyses showed that HORMS aberrations were significantly lower in the EDOF group compared to the control group (*p* < 0.001). Reduced spherical aberration, in particular, is beneficial for not introducing severe optical side effects. The residual spherical aberration in the EDOF group was significantly lower (−0.005 ± 0.038 µm) than the monofocal (0.036 ± 0.036 µm; *p* < 0.001). Coma aberration remained similar between groups, indicating that no additional asymmetric aberrations were induced.

### 4.5. Patient-Reported Outcomes

Patient-reported outcomes are crucial for evaluating subjective satisfaction and quality of life after IOL implantation. In this study, the Catquest-9SF questionnaire showed that 70.59% of patients in the Asqelio™ EDOF group were “satisfied” or “very satisfied” with their postoperative vision. None reported being “very unsatisfied”, suggesting that the majority of patients found the visual performance acceptable, if not excellent. Daily activities, especially those requiring intermediate vision, were reported as easy to perform by most patients. Reading small print or newspapers poses a significant challenge for roughly a third of patients, a known limitation when extended ranges of focus prioritize intermediate and distance vision over near tasks. Visual symptoms, including glare, halos, starbursts, and blurred vision, can negatively impact patient satisfaction even if visual acuity measures are excellent. However, the present study’s symptom questionnaire revealed low frequency, intensity, and bothersomeness of such phenomena. The low incidence of disturbing photic phenomena aligns with other literature indicating that non-diffractive EDOF IOLs induce fewer dysphotopsias compared to trifocal or diffractive EDOF lenses [15,22]. Other studies of other non-diffractive wavefront-shaping EDOF IOLs also report minimal differences in visual disturbances compared to monofocals [14,18], supporting the notion that EDOF technology can deliver a balance between extended range of vision and quality of vision.

Regarding posterior capsule opacification (PCO), contemporary hydrophobic acrylic IOLs have been shown to exhibit a very low incidence of PCO in the years following cataract surgery. Large real-world studies report that 3–5 years after surgery, eyes with modern hydrophobic acrylic IOLs develop PCO at only single-digit percentage rates—on the order of ~5%—which is dramatically lower than the PCO incidence observed with earlier-generation or hydrophilic acrylic lenses [23]. In our study, no clinically significant PCO was observed during the follow-up period, although the short duration of follow-up limits the assessment of such events

The selection of IOLs has become more challenging as surgeons must consider a patient’s lifestyle, expectations, and ocular health. Non-diffractive EDOF IOLs like Asqelio™ EDOF offer an appealing compromise: better intermediate vision than monofocals and fewer visual disturbances than diffractive multifocals or trifocals, though near reading tasks may still require additional correction. Recent consensus statements and Delphi panels have emphasized the need for individualized patient counseling and thorough preoperative assessments to predict satisfaction and reduce postoperative dissatisfaction [24]. Incorporating mini-monovision strategies may further enhance near vision and patient independence from reading glasses.

## 5. Conclusions

The Asqelio™ EDOF IOL provided good refractive predictability, effective uncorrected vision across distance and intermediate ranges, and high patient satisfaction with minimal visual disturbances, indicating good subjective quality of vision. The contrast sensitivity and optical scatter outcomes were comparable to monofocal implants, and the lower HOARMS and spherical aberration in the Asqelio™ EDOF IOL group compared to the control monofocal group suggest good optical quality of the retinal image. We estimate that this IOL can be considered a valuable option for patients seeking an extended range of functional vision with minimal side effects. Future studies with longer follow-up periods and large samples should be performed. These investigations should also explore the toric version of this model.

## Figures and Tables

**Table 1:** Descriptive statistics (mean ± standard deviation) of the study sample.

	EDOF IOL Group	Control IOL Group	*p*-Value
Age (y)	64.21 ± 8.24	73.45 ± 5.87	<0.001
Sphere (D)	0.93 ± 2.62	1.12 ± 2.05	0.369
Refractive Cylinder (D)	0.61 ± 0.59	0.47 ± 0.41	0.118
Spherical Equivalent (D)	0.63 ± 2.72	0.89 ± 2.07	0.328
CDVA (logMAR)	0.25 ± 0.38	0.19 ± 0.15	0.272
K1 (D)	43.19 ± 1.36	43.59 ± 1.56	0.181
K2 (D)	43.70 ± 1.37	44.10 ± 1.62	0.213
Axial length (mm)	23.22 ± 1.29	23.39 ± 1.01	0.262
ACD (mm)	3.05 ± 0.37	3.08 ± 0.32	0.369
LT (mm)	4.55 ± 0.43	4.65 ± 0.34	0.135
WTW (mm)	11.97 ± 0.29	12.03 ± 0.31	0.220
IOL spherical power (D)	22.95 ± 3.08	21.93 ± 2.65	0.008

IOL: intraocular lens; CDVA: corrected distance visual acuity; K1: flat corneal meridian; K2: steep corneal meridian; ACD: anterior chamber depth; LT: lens thickness; WTW: white-to-white distance.

**Table 2:** LogMAR visual acuity outcomes of patients implanted with the Asqelio™ EDOF intraocular lens shown as means, standard deviations (SD), and ranges.

	Mean ± SD (Range)
Monocular Photopic UDVA	0.08 ± 0.13 (−0.10 to 0.40)
Binocular Photopic UDVA	0.00 ± 0.09 (−0.22 to 0.20)
Monocular Photopic CDVA	0.00 ± 0.09 (−0.20 to 0.20)
Binocular Photopic CDVA	−0.03 ± 0.08 (−0.20 to 0.20)
Monocular Photopic UIVA	0.19 ± 0.13 (0.00 to 0.60)
Binocular Photopic UIVA	0.13 ± 0.12 (−0.10 to 0.50)
Monocular Photopic CDIVA	0.13 ± 0.09 (0.00 to 0.30)
Binocular Photopic CDIVA	0.09 ± 0.08 (−0.10 to 0.20)
Monocular Photopic UNVA	0.41 ± 0.17 (0.10 to 0.90)
Binocular Photopic UNVA	0.32 ± 0.15 (0.10 to 0.70)
Monocular Photopic CDNVA	0.39 ± 0.13 (0.10 to 0.60)
Binocular Photopic CDNVA	0.29 ± 0.11 (0.10 to 0.50)

UDVA: uncorrected distance visual acuity; CDVA: corrected distance visual acuity; UIVA: uncorrected distance intermediate visual acuity; CDIVA: corrected distance intermediate visual acuity; UNVA: uncorrected distance near visual acuity; CDNVA: corrected distance near visual acuity.

**Table 3:** Contrast sensitivity and optical quality outcomes of patients implanted with the EDOF and control intraocular lens groups shown as means and standard deviations.

	EDOF Group	Control Group	*p*-Value
Contrast sensitivity (log units)	1.67 ± 0.10	1.66 ± 0.23	*p* = 0.386
Ocular dispersion index	1.73 ± 0.77	1.52 ± 0.82	*p* = 0.160
Higher order aberrations RMS (μm)	0.171 ± 0.063	0.329 ± 0.171	*p* < 0.001
Coma aberration (μm)	0.115 ± 0.061	0.095 ± 0.058	*p* = 0.074
Spherical aberration (μm)	−0.005 ± 0.038	0.036 ± 0.036	*p* < 0.001

**Table 4:** Summary of patient-reported difficulties and satisfaction with their vision as per Catquest-9SF.

Do You Have Difficulty…	Response Frequencies (%)				
	Yes, Extreme Difficulty	Yes, Great Difficulty	Yes, Some Difficulty	No, No Difficulty	Cannot Decide
…Reading text in newspapers?	0.00	29.41	35.29	35.29	0.00
…Recognizing the faces of people you meet?	0.00	5.88	17.65	76.47	0.00
…Seeing the prices of goods when shopping?	0.00	11.76	41.18	47.06	0.00
…Seeing to walk on uneven surfaces?	0.00	11.76	5.88	82.35	0.00
…Seeing to do handicrafts, woodwork etc.?	0.00	17.65	23.53	29.41	29.41
…Reading subtitles on TV?	5.88	11.76	29.41	52.94	0.00
…Seeing to engage in an activity/hobby?	0.00	17.65	41.18	41.18	0.00

**Table 5:** Summary of patient reported visual symptoms (type of symptom and frequency of responses). Response coding (frequency/severity/bothersome): R1 (never/none/none), R2 (occasionally/mild/a little), R3 (quite often/moderate/quite a bit), R4 (very often/severe/a lot).

	Frequency (%)					Frequency (%)			
	R1	R2	R3	R4		R1	R2	R3	R4
Glare					Distorted vision				
Frequency	50.0	44.4	5.6	0.0	Frequency	66.7	27.8	5.5	0.0
Intensity	55.5	33.3	11.1	0.0	Intensity	66.7	22.2	11.1	0.0
Bothersome	61.1	38.9	0.0	0.0	Bothersome	66.7	27.8	0.0	5.5
Halo					Fluctuation in vision				
Frequency	61.1	38.9	0.0	5.5	Frequency	88.9	5.5	0.0	5.5
Intensity	61.1	33.3	5.5	0.0	Intensity	88.9	5.5	5.5	0.0
Bothersome	66.7	33.3	0.0	0.0	Bothersome	88.9	5.5	5.5	0.0
Starburst					Difficulty focusing				
Frequency	77.8	22.2	0.0	0.0	Frequency	55.5	38.9	0.0	5.5
Intensity	77.8	16.7	5.5	0.0	Intensity	55.5	38.9	0.0	5.5
Bothersome	77.8	22.2	0.0	0.0	Bothersome	61.1	33.3	0.0	5.5
Hazy vision					Difficulty focusing				
Frequency	72.2	22.2	0.0	5.5	Frequency	61.1	27.8	0.0	11.1
Intensity	77.8	11.1	5.5	5.5	Intensity	61.1	27.8	5.5	5.5
Bothersome	72.2	22.2	0.0	5.5	Bothersome	72.2	16.7	5.5	5.5
Blurred vision					Difficulty perceiving distances/depth				
Frequency	55.5	16.7	11.1	16.7	Frequency	72.2	16.7	5.5	5.5
Intensity	55.5	16.7	27.8	0.0	Intensity	72.2	11.1	5.5	11.1
Bothersome	66.7	22.2	5.5	5.5	Bothersome	72.2	16.7	0.0	11.1

## Data Availability

Anonymized data from the study may be made available upon reasonable request to the corresponding author, subject to a formal request and a justified purpose.
